# Inheritance and Characterization of Strong Resistance to Phosphine in *Sitophilus oryzae* (L.)

**DOI:** 10.1371/journal.pone.0124335

**Published:** 2015-04-17

**Authors:** Tam T. Nguyen, Patrick J. Collins, Paul R. Ebert

**Affiliations:** 1 School of Biological Sciences, University of Queensland, St. Lucia, Queensland, Australia; 2 Faculty of Biology, Hanoi National University of Education, Hanoi, Vietnam; 3 Department of Agriculture, Fisheries and Forestry, Ecosciences Precinct, Brisbane, Queensland, Australia; University of California, Berkeley, UNITED STATES

## Abstract

*Sitophilus oryzae* (Linnaeus) is a major pest of stored grain across Southeast Asia and is of increasing concern in other regions due to the advent of strong resistance to phosphine, the fumigant used to protect stored grain from pest insects. We investigated the inheritance of genes controlling resistance to phosphine in a strongly resistant *S*. *oryzae* strain (NNSO7525) collected in Australia and find that the trait is autosomally inherited and incompletely recessive with a degree of dominance of -0.66. The strongly resistant strain has an LC50 52 times greater than a susceptible reference strain (LS2) and 9 times greater than a weakly resistant strain (QSO335). Analysis of F2 and backcross progeny indicates that two or more genes are responsible for strong resistance, and that one of these genes, designated *So_rph1*, not only contributes to strong resistance, but is also responsible for the weak resistance phenotype of strain QSO335. These results demonstrate that the genetic mechanism of phosphine resistance in *S*. *oryzae* is similar to that of other stored product insect pests. A unique observation is that a subset of the progeny of an F1 backcross generation are more strongly resistant to phosphine than the parental strongly resistant strain, which may be caused by multiple alleles of one of the resistance genes.

## Introduction

Fumigation is the most widely used method of protecting stored grain against insect pests, as it is readily applied to all types of storages including silos, warehouses, bunkers, bag stacks, ships during transport, and cereal mills [1]. Phosphine (PH_3_) is an ideal fumigant to disinfest bulk commodities as it is cost-effective, penetrates grain bulks readily, does not leave residues and can be rapidly eliminated from the grain via aeration [2]. Alternative fumigants are limited as use of methyl bromide for routine grain fumigation has been phased out in developed countries since 2005 and will only be allowed in developing countries until 2015 [1]. Whilst, sulphuryl fluoride is only accepted for treatment of grain in some nations as there are concerns about potential fluoride residues [1, 3]. The lack of practicable alternatives has placed very heavy reliance on phosphine for fumigation and this has inevitably resulted in selection for resistance to this fumigant in many insect pests of stored products [4].

Resistance toward phosphine in grain pests was first reported in a survey of insecticide resistance in insects from many countries conducted in 1972–1973. This report concluded that *Sitophilus* spp. including *Sitophilus oryzae* were the greatest threat to postharvest agricultural products in terms of resistance to insecticides. At that time, phosphine resistance in *S*. *oryzae* was found in only 5% of tested samples [5]. However, by 2000, the frequency of resistance in this species had increased sharply to 75% of samples in developing countries and reached a peak of 100% in Brazil [4]. Resistance to phosphine in insect pests of stored grain has now become a serious international problem, and as there is no practical replacement for this fumigant in most cases, developing strategies to manage the problem is a priority. A rational approach to manage this resistance, however, requires an understanding of the underlying genetic mechanisms controlling this phenomenon. Available information, however, is incomplete. Two genetic studies of resistance to phosphine induced mortality in *S*. *oryzae* have been carried out. Resistance in *S*. *oryzae* collected in China is autosomal, incompletely recessive and conferred by more than one gene [6]. Recently, Daglish [7] reported weak resistance of an Australian strain being autosomal, incompletely recessive and monogenic.

Strong resistance to phosphine was first reported in *S*. *oryzae* in China in a 1995–1997 survey in which a resistance level 337 times that of a fully susceptible strain was observed [8]. The resistance level of this species in India was reported in 1998 to have increased to 425 times that of a susceptible reference strain [9].Weakly resistant *S*. *oryzae* is found at a high frequency in most regions of Australia, with strong resistance occurring sporadically in field collected strains [10].

Studies on the molecular genetics of resistance to phosphine have been carried out on *Rhyzopertha dominica* and *Tribolium castaneum*. In these species, two loci act synergistically to cause strong resistance. The first gene (*rph1 or tc_rph1*) is responsible for weak resistance but its identity is currently unknown, whereas the second gene (*rph2 or tc_rph2*) was determined to be dihydrolipoamide dehydrogenase (dld) in both species [11, 12]. Individuals that are homozygous for resistance alleles of either *rph1* or *rph2* are weakly resistant, whereas individuals that are homozygous for resistance alleles at both loci are strongly resistant to phosphine [13, 14]. The growing problem of resistance in *S*. *oryzae* and the pressing need to manage this resistance, together with the recent identification of the *rph2* resistance gene, led to our aim of determining the mechanism of inheritance of the strong resistance trait in this species.

## Materials and Methods

### Insect strains: origin and culture

A laboratory susceptible strain and two phosphine resistant strains of *S*. *oryzae*, one weak and one strong, were used in this study. The susceptible strain (LS2), denoted S-strain in this report, was collected from Brisbane (27°27'59.7"S 153°01'19.3"E) in south-east Queensland in 1965 [7, 15] and has been maintained under pesticide free conditions since that time. The weakly resistant strain (QSO335), denoted W-strain, was collected from a central grain storage in Millmerran (27°52'22.4"S 151°16'07.5"E), south-east Queensland in 1990 [7, 15], while the strongly resistant strain (NNSO7525), called R-strain in this report, was collected in 2009 from a farm at Widgelli (34°19'46.9"S 146°08'14.8"E), near Griffith in southern New South Wales. The resistant strains were exposed to phosphine at 0.25 mgL^-1^ (for R-strain) and at 0.04 mgL^-1^ (for W-strain) for 48 h to select individuals homozygous for the resistance alleles. This selection process was repeated on at least 3 generations for each strain. All insects were cultured on whole wheat at 25°C and 55% relative humidity (RH).

### Genetic crosses

Mass mated reciprocal crosses of either the S-strain or the W-strain with the R-strain were established by combining 50 virgin adult males of one strain with 50 virgin females of another strain. Every two weeks, the P_0_ parents were transferred to fresh grain. After eight weeks, the two to four week old F_1_ of each cross was transferred to fresh food to produce the F_2_ generation. The F_1_ and F_2_ generations were subsequently tested for their response to phosphine.

### Backcross of F_1_ offspring to the strongly resistant parental strain

Virgin F_1_ females produced from crosses (♀ S-strain x ♂ R-strain, n = 50) and (♀W-strain x ♂R-strain, n = 50) were each mated with 50 males of the parental R-strain to generate a backcross (F_1_-BC). Insects were fed on whole wheat and incubated at 25°C and 55% RH. At 2–4 weeks post-emergence, adults of each F_1_-BC were fumigated with phosphine.

### Phosphine fumigation

Phosphine fumigation was carried out based on the recommended FAO method for testing resistance [16]. Phosphine gas was generated from commercial formulations of aluminium phosphide in a 5% solution of sulphuric acid. The concentration of the generated phosphine was measured using a gas chromatograph fitted with a thermal conductivity detector (TCD) (Perkin and Elmer, Clarus 580).

We used 100–150 adults (2–4 weeks old) for each dosage level tested as well as a control, which did not receive a phosphine dosage. Test insects were placed into plastic cups with small holes in their lids without food. These were then placed into gas-tight desiccators and phosphine was injected into the desiccators (except for control desiccator) through a septum. Insects were exposed to phosphine for 48 h at 25°C and 50% RH. After fumigation, insects were placed on whole wheat and allowed to recover for seven days at 25°C and 55% RH, at which time mortality was assessed. Each fumigation was undertaken three separate times at three week intervals.

Hybrid generations (i.e. the F_1_, F_2_ or F_1_-BC) and their parental strains were fumigated simultaneously with phosphine at a range of doses. Concentrations of phosphine used for fumigation ranged from 0.002 to 0.03 mgL^-1^ for the S-strain; from 0.008 to 0.15 mgL^-1^ for the W-strain; and from 0.1 to 1.0 mgL^-1^ for the R-strain. F_1_ hybrids of the S-strain x R-strain cross were fumigated from 0.003 to 0.04 mgL^-1^, while F_1_ hybrids of the W-strain x R-strain cross were exposed to doses of 0.02 to 0.3 mgL^-1^. The F_2_ (S-strain x R-strain) was fumigated with a range of doses from 0.005 to 0.4 mgL^-1^, and the F_2_ progeny of the W-strain x R-strain cross were fumigated from 0.02 to 0.6 mgL^-1^. The F_1_ (♀S-strain x ♂R-strain)-BC and F_1_ (♀W-strain x ♂R-strain)-BC were exposed to phosphine from 0.005 to 0.9 mgL^-1^ and from 0.03 to 0.9 mgL^-1^, respectively.

### Data analysis

The responses to phosphine of the parental strains and the reciprocal F_1_ progenies were analyzed by probit regression [17] using GenStat 11.1 software [18]. Abbott’s formula [19] was used to correct for mortality observed in control insects, which never exceeded 3%. The probit analysis was used to determine the genetic homogeneity of the parental strains and the resulting F_1_ progeny, the expected mortality based on a single gene model of resistance, the observed LC_50_s and whether the responses of the reciprocal F_1_ populations differed from each other.

### Inheritance of strong resistance to phosphine

The parental strains (P_0_) and their respective F_1_ progenies were initially tested for genetic uniformity of resistance by calculating the heterogeneity factor (hf) and evaluating whether chi square values (χ^2^) revealed a significant difference between observed responses and expected values derived from the probit model. Doses at which fewer than 5 individuals were expected either to live or to die were excluded from the chi square analysis as suggested by Finney [17]. Resistant parental strains underwent multiple rounds of phosphine selection at the relevant discriminating dose to promote homozygosity at the resistance loci, hence the response of F_1_ generations were expected to be genetically homogeneous. On this basis, the expected mortality of the P_0_ and F_1_ at each dose was calculated by the linear equation: *y* = *α* + *βx*, in which *y* is mortality at *x* concentration and *α*, *β* are the intercept and slope, respectively. Chi-square and degrees of freedom were then determined according to Finney [17] using observed and expected data.

Resistance factors at 50% mortality between the S-strain and the W-strain and between the S-strain and the R-strain were determined according to Robertson et al., 2007 [20]. The reciprocal F_1_s were judged not to be significantly different from each other as the 95% confidence limits of the relative potency analysis included 1. The method developed by Stone [21] was used to calculate the degree of dominance (D). Degree of dominance ranges from -1 to +1 where -1 is completely recessive and +1 is completely dominant.

### Gene(s) responsible for strong resistance to phosphine

The number of genes contributing to strong resistance was determined from analyses of the responses of the F_2_ progeny derived from a cross between the S-strain and the R-strain and the progeny of their F_1_ backcrossed (F_1_-BC) to the R-strain. In a similar way, the W-strain was also crossed with the R-strain to identify genetic differences between these two phenotypes. In each case, the actual mortality was compared to the predicted mortality at each experimentally tested dose (x) based on the hypotheses that a single gene is responsible for the difference between susceptible and strongly resistant insects or that a single gene is responsible for the difference between weakly resistant and strongly resistant insects. Based on segregation ratios of genotypes at F_2_ and F_1_-BC generations from the single gene model, the predicted mortality at each dose of the F_2_ (W_x_ (F_2_)) was calculated using the equation Wx(F2) = W(SS) * 25 + W(RS) * 50 + W(RR) * 25, and the predicted mortality of the F_1_-BC calculated as Wx(F1−BC) = W(RS) * 50 + W(RR) * 50, where the mortality factors W(SS), W(RS), W(RR) are estimated from the probit model of the responses to phosphine of the S-strain, F_1_ hybrids and the R-strain, respectively [22]. The statistical difference between observed and predicted data was evaluated using the modified chi-square method (modified χ^2^) [23]. The modified χ^2^ analysis incorporates a weighted mean heterogeneity factor calculated from all strains contributing to the genetic composition of the F_2_ or F_1_-BC [23]. Modified χ^2^ values indicate no significant difference between theoretical and experimental mortalities if the probability value (*P*) is greater than 5% (*P* > 0.05) [17]. We also compared the shapes of the experimentally determined and expected response curves of the F_2_ and F_1_-BC populations to test the mode of inheritance of the resistance trait. The expectation, if a single gene is responsible for the increase in resistance, is for a plateau in the response curve of the F_2_ at 25% mortality if the resistance trait is dominant and 75% if it is recessive. Similarly, a plateau at 50% mortality is expected in the case of the F_1_-BC [24].

## Results

### Analysis of the S-strain x R-strain cross

#### Mode of inheritance of strong resistance to phosphine

**Fig 1:**
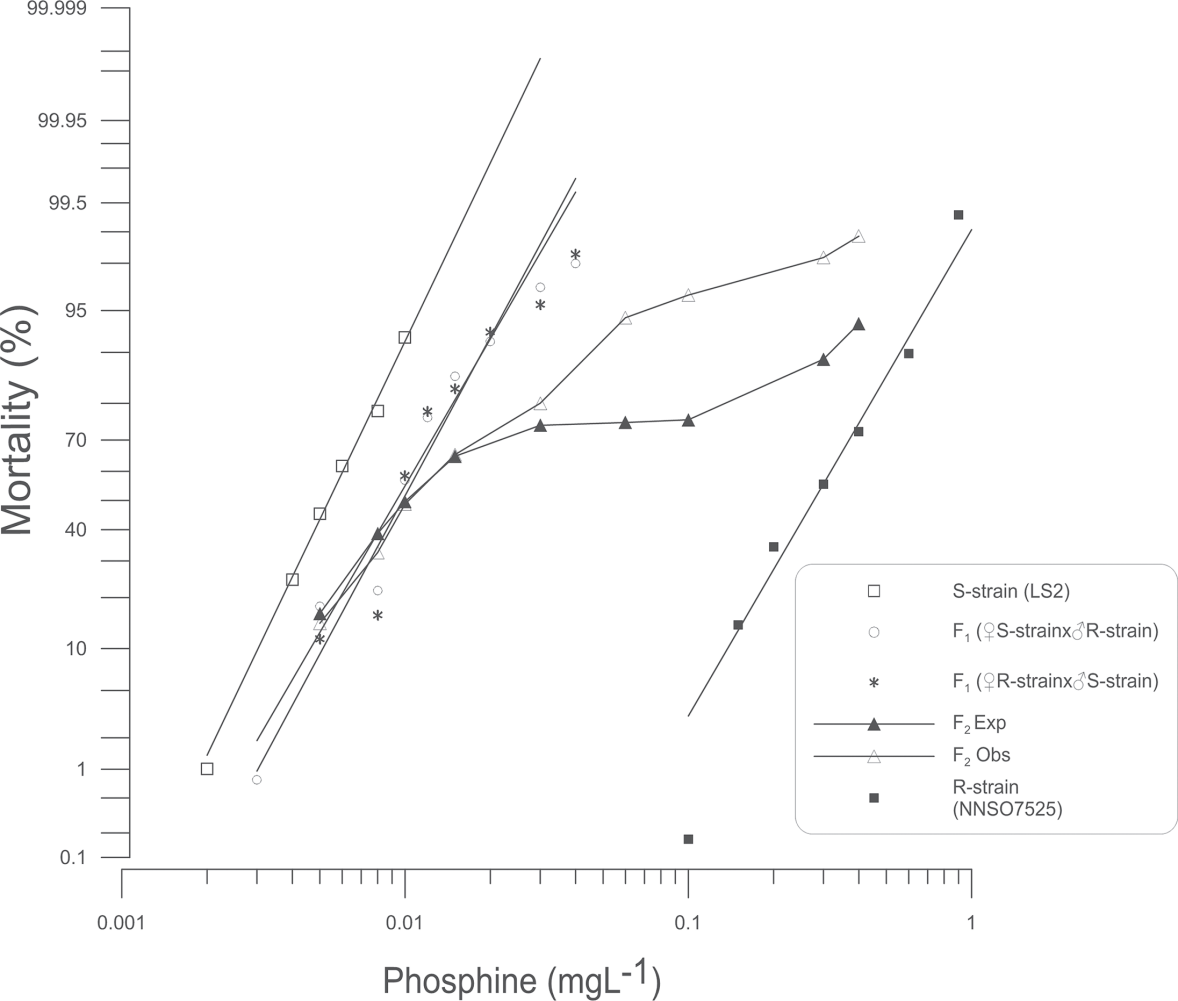
Probit analysis of mortality due to phosphine exposure: S-strain x R-strain intercross. Percent mortality was determined after a 48 h exposure to phosphine at 25°C followed by a week recovery period. Results for susceptible (S-strain) and strongly resistant (R-strain) insects are provided for reference. Experimental data for reciprocal F_1_ and F_2_ progeny are shown. A theoretical mortality response curve for the F_2_ is drawn based on the hypothesis that only a single gene contributes to the observed resistance.

The probit curve of the S-strain indicated that it was genetically homogeneous with respect to its response to phosphine (*P* = 0.121) (Table 1). This genetic uniformity was clearly reflected in the log dose-probit mortality (ld-pm) line, which showed that observed plots of the S-strain matched the probit model exactly (Fig 1). In contrast, the strongly resistant (R-strain) and the reciprocal F_1_ progeny exhibited heterogeneity (*P* < 0.001 and hf = 8.55, 16.49, 21.89 for the R-strain, F_1SR,_ F_1RS_, respectively) (Table 1). The level of resistance of the R-strain was calculated at the LC_50_ to be 52× that of the reference S-strain, and 2× for both reciprocal F_1_ progenies. The degree of dominance of F_1_(♀S-strain x ♂R-strain) and F_1_(♀R-strain x ♂S-strain) were -0.71 and -0.66, respectively (Table 1), indicating that the strong resistance trait was incompletely recessive. The ld-pm lines of the reciprocal F_1_ populations were essentially collinear (Fig 1) and the relative potency of F_1_(♀S-strain x ♂R-strain) and F_1_(♀R-strain x ♂S-strain) was 1.04 (0.866–1.250, 95% CL), providing reliable confirmation that the response to phosphine of each of the reciprocal F_1_ populations were indistinguishable. This proved the absence of maternal effects, indicating that strong resistance in *S*. *oryzae* is autosomal, and that the data from the reciprocal F_1_s could be pooled in later analyses.

**Table 1:** Analysis of heterogeneity and strength of the phosphine resistance trait, as well as the degree of dominance of the reciprocal F_1_ progeny of a S-strain x R-strain cross.

Strain/cross	n	Slope (± SE)	LC_50_ (95%FL) (mgL^-1^)	hf	df	χ^2^	P	LC_50_ ratio (95% CL)	DD
**S-strain**	3891	5.13 (± 0.16)	0.005 (0.005–0.006)	1.83	4	7.30	0.121	-	-
**R-strain**	4223	4.21 (± 0.33)	0.277 (0.245–0.308)	8.55	4	34.18	6.84E-07***	52 (27.23–97.45)	-
**F**_**1**_ **(♀S x ♂R)**	3752	4.22 (± 0.50)	0.009 (0.008–0.010)	16.49	6	98.95	4.16E-19***	2 (1.36–2.22)	-0.71
**F**_**1**_ **(♀R x ♂S)**	3343	4.56 (± 0.66)	0.01 (0.008–0.011)	21.89	6	131.34	6.71E-26***	2 (1.34–2.46)	-0.66
**F**_**1**_ **pooled**	7095	4.37 (± 0.38)	0.01 (0.009–0.010)	18.23	12	218.73	4.35E-40***	2 (1.36–2.32)	-0.66

n = number of insects tested; SE = standard error; LC_50_ = lethal concentration at 50%; CL = confidence limit; FL = fiducial limit; hf = heterogeneity factor; df = degree of freedom; χ^2^ = chi-square; P = probability value; DD = degree of dominance;

*Significant (P<0.05);

**Significant (P<0.01);

***Significant (P<0.001).

#### Gene(s) controlling strong resistance

As the resistant trait was incompletely recessive, the hypothesis of monogenic inheritance predicts a plateau in the response line of the F_2_ at 75% mortality. In the F_2_ progeny of the cross between the S-strain and the R-strain, there was no significant difference (*P*>0.05) between expected and observed data at low to intermediate doses from 0.005 to 0.03 mgL^-1^ (S1 Table) and a shoulder was indeed observed at 75% mortality (from 0.15 to 0.03mgL^-1^) (Fig 1). However, at doses from 0.06 to 0.3 mgL^-1^, the observed mortality was significantly greater than the response predicted by the monogenic inheritance model (*P* = 9.5E-05, 2.79E-05, 0.0093 with df = 1) (S1 Table).

**Fig 2:**
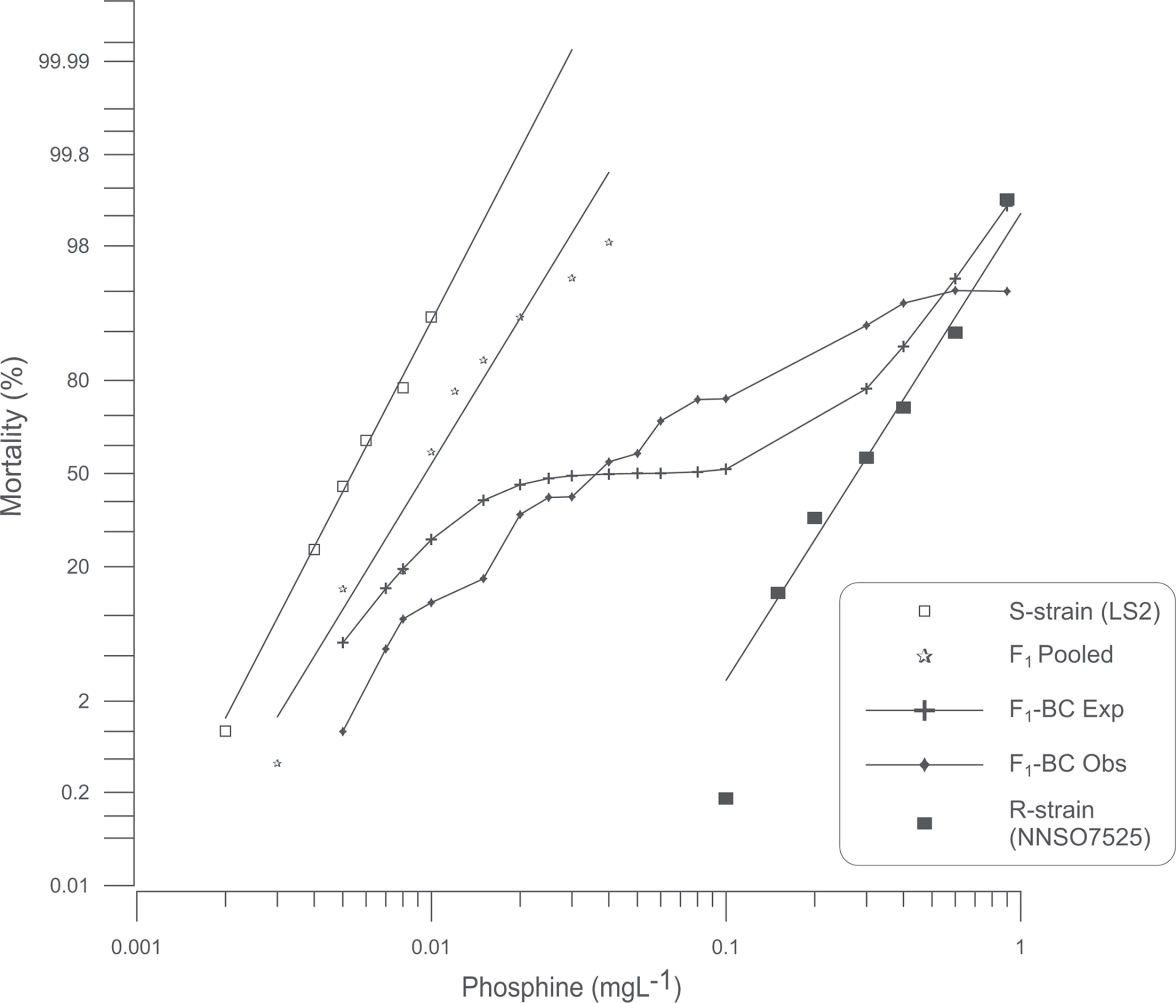
Probit analysis of mortality due to phosphine exposure: S-strain x R-strain backcross. Percent mortality was determined after a 48 h exposure to phosphine at 25°C followed by a week recovery period. Results for susceptible (S-strain) and strongly resistant (R-strain) insects are provided for reference. Experimental data for pooled F_1_ progeny as well as the progeny of the backcross of the F_1_ to the R-strain parent are shown. A theoretical mortality response curve for the F_1_-BC is drawn based on the hypothesis that only a single gene contributes to the observed resistance.

The monogenic hypothesis predicts a plateau in the response curve of F_1_-BC at 50% mortality. However, in the backcross of F_1_(S-strain x R-strain) to R-strain, there was evidence for at least three plateaus appearing at mortality levels of 42%,75% and 95% instead of one plateau at 50% as expected (Fig 2). Modified chi-square values showed significant deviations across the range of tested doses at a moderately low dose (*P* = 0.019), moderately high doses (*P* = 0.040, 0.030 and 0.008) and an extremely high dose (*P* = 0.007) (S2 Table). Notably, observed mortality was less than expected at the moderately low dose, but greater than expected at moderately high doses. Thus, it appears that more than one factor, potentially with unequal effects, contribute to the strong resistance phenotype [25], however, this interpretation leaves the plateau at 95% mortality unexplained. The responses of the S-strain x R-strain cross and backcross progeny provide strong evidence that the monogenic hypothesis can be rejected, confirming that more than one gene controls strong resistance to phosphine.

### Reciprocal crosses between the W-strain and the R-strain

#### Dominance, resistance factor and sex-linkage of resistance to phosphine

**Fig 3:**
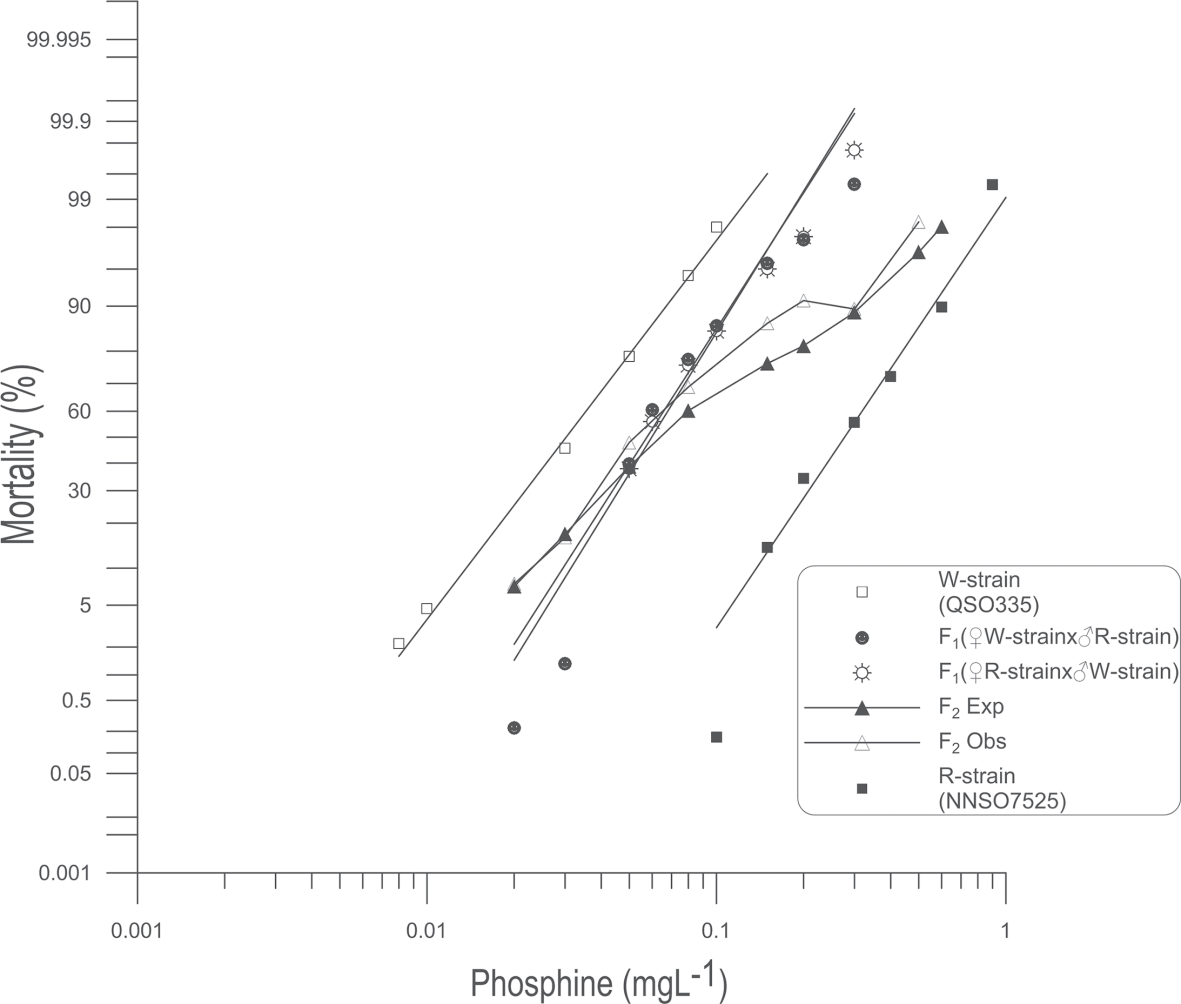
Probit analysis of mortality due to phosphine exposure: W-strain x R-strain intercross. Percent mortality was determined after a 48 h exposure to phosphine at 25°C followed by a week recovery period. Results for weakly resistant (W-strain) and strongly resistant (R-strain) insects are provided for reference. Experimental data for reciprocal F_1_ and F_2_ progeny are shown. A theoretical mortality response curve for the F_2_ is drawn based on the hypothesis that only a single gene contributes to the observed resistance.

The response of W-strain to phosphine (Table 2) indicated that it was homogeneous (*P* = 0.068), whereas those of the R-strain, F_1_ (♀W-strain x ♂R-strain) and F_1_ (♀R-strain x ♂W-strain) exhibited considerable heterogeneity (*P* < 0.001 and hf = 8.55, 6.94, 6.42, respectively). The resistance at the LC_50_ of the W-strain was 9 times less than that of the R-strain but 6 times greater than that of the S-strain. The degree of dominance based on the response of the F_1_ (♀W-strain x ♂R-strain) and F_1_ (♀R-strain x ♂W-strain) progeny was -0.38 and -0.41, respectively (Table 2), indicating that the strong resistance trait was incompletely recessive. The ld-pm lines of the reciprocal F_1_ hybrids overlapped (Fig 3) and the relative potency between F_1_ (♀W-strain x ♂R-strain) and F_1_ (♀R-strain x ♂W-strain) was 1.033 [0.9023–1.184, 95%CL], showing that two data sets of the reciprocal F_1_ populations were statistically indistinguishable. Thus, strong resistance relative to weak resistance to phosphine in *S*. *oryzae* was neither sex-linked nor maternally influenced, allowing data of reciprocal F_1_ crosses to be combined in the subsequent analysis.

**Table 2:** Analysis of heterogeneity and strength of the phosphine resistance trait, as well as the degree of dominance of the reciprocal F_1_ progeny of a W-strain x R-strain cross.

Strain/cross	n	Slope (± SE)	LC_50_ (95%FL) (mgL^-1^)	hf	df	χ^2^	P	LC_50_ ratio (95% CL)	DD
**W-strain**	4202	3.71 (± 0.15)	0.030 (0.028–0.033)	2.18	4	8.73	0.068	-	-
**R-strain**	4223	4.21 (± 0.33)	0.277 (0.245–0.308)	8.55	4	34.18	6.84E-07***	9 (4.76–17.63)	-
**F**_**1**_ **(♀R x ♂W)**	3353	4.59 (±0.45)	0.060 (0.053–0.067)	6.42	5	32.10	5.68E-06***	2 (1.35–2.89)	-0.38
**F**_**1**_ **(♀W x ♂R)**	3757	4.42 (± 0.44)	0.058 (0.051–0.064)	6.94	5	34.70	1.73E-06***	2 (1.30–2.78)	-0.41
**F**_**1**_ **pooled**	7110	4.50 (± 0.30)	0.059 (0.055–0.063)	9.74	10	97.37	1.83E-16***	2 (1.33–2.83)	-0.39

n = number of insects tested; SE = standard error; LC_50_ = lethal concentration at 50%; CL = confidence limit; FL = fiducial limit; hf = heterogeneity factor; df = degree of freedom; χ^2^ = chi-square; P = probability value; DD = degree of dominance;

*Significant (P<0.05);

**Significant (P<0.01);

***Significant (P<0.001).

#### Gene(s) responsible for strong resistance as determined from weak and strong resistance strains

Our analysis of the cross between the S-strain and the R-strain suggested that two or more incompletely recessive genes contribute to the strong resistance phenotype. Previous research indicated that one incompletely recessive gene is responsible for weak resistance in the W-strain [7]. The implication is that strong resistance is due to the effect of the gene responsible for weak resistance as well as one or more additional genes. We analyzed the progeny of a cross between the W-strain and the R-strain to determine whether the resistance factor in the W-strain is also found in the R-strain, as well as to determine the number of additional genes that contribute to resistance in the R-strain.

If there is no common resistance gene shared between the weakly and strongly resistant strains, their F_1_ progeny will be heterozygous at each resistance locus. As a result, the dominant susceptibility phenotype will be expressed and the F_1_ progeny will be almost completely sensitive to phosphine. This is not the case, as the observed mortality curve of the F_1_ progeny indicated a higher level of resistance than the W-strain (Fig 3). The simplest explanation for the result is that the resistance factor in the W-strain is also present in the R-strain and that one or more additional factors are responsible for the greater level of resistance in the R-strain. The fact that the probit curve of the F_1_ was closest to the curve of W-strain rather than the strong resistance R-strain (Fig 3) and the degree of dominance of pooled F_1_ determined to be -0.39 (Table 2) indicate that the additional resistance factor in the R-strain is incompletely recessive. The resistance levels of the reciprocal F_1_ progeny were about twice that of the W-strain.

If the additional resistance phenotype of the R-strain is contributed by a single gene, 25% of the F_2_ progeny will be homozygous recessive and therefore strongly resistant. Because the degree of resistance between the R-strain and the W-strain was not very great (i.e. only 9×) and the resistance was only incompletely recessive, a weak inflection of the F_2_ response curve at 75% mortality can be anticipated, rather than a distinct plateau [24]. It was clear from results shown in Fig 3 and S3 Table, however, that there was no inflection observed within the expected dose range (0.1–0.15 mgL^-1^) but the actual plateau was at 91% mortality, and the observed mortality was significantly greater than expected in response to phosphine from 0.05 to 0.2 mgL^-1^(*P* < 0.05 and *P* < 0.01).

**Fig 4:**
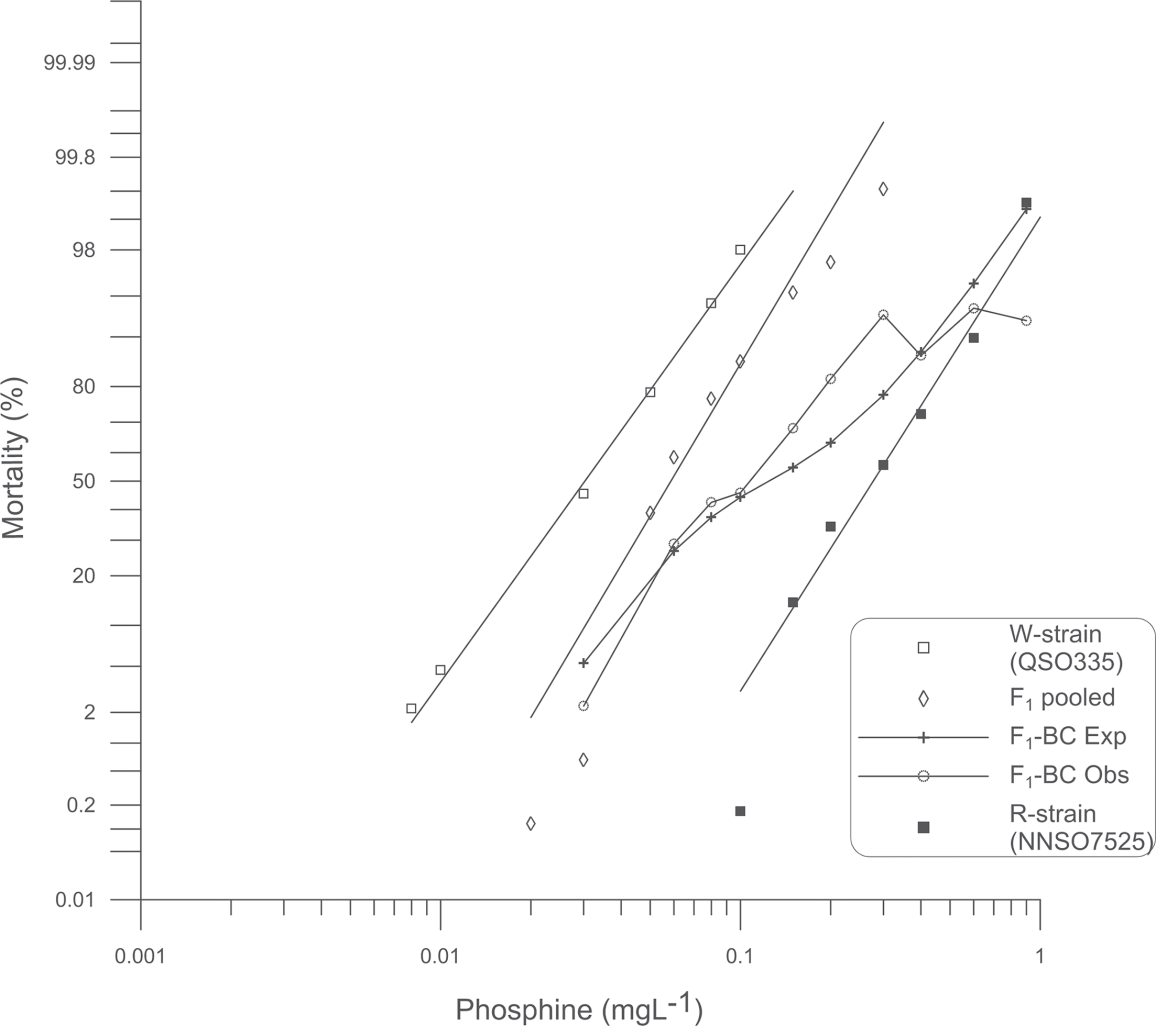
Probit analysis of mortality due to phosphine exposure: W-strain x R-strain backcross. Percent mortality was determined after a 48 h exposure to phosphine at 25°C followed by a week recovery period. Results for weakly resistant (W-strain) and strongly resistant (R-strain) insects are provided for reference. Experimental data for pooled F_1_ progeny as well as the progeny of the backcross of the F_1_ to the R-strain parent are shown. A theoretical mortality response curve for the F_1_-BC is drawn based on the hypothesis that only a single gene contributes to the observed resistance.

To test the hypothesis that the difference between weak and strong resistance is a monogenic trait, the F_1_ progeny of a cross between the W-strain (♀) and the R-strain (♂) was backcrossed to the R-strain. A monogenic hypothesis predicts that half of the progeny of the F_1_-BC will be homozygous resistant, whereas the other half will be heterozygous for the additional gene. Thus an inflection in the response curve of the F_1_-BC progeny is expected at 50% mortality. A shoulder was indeed observed at 42–45% mortality (Fig 4). However, this was followed by significantly greater than expected mortality in response to 0.2 mgL^-1^ and 0.3 mgL^-1^ phosphine (*P* < 0.01) (S4 Table). Interestingly, a very distinct plateau occurred at 93% mortality that persisted through the highest dose tested, 0.9 mgL^-1^, which was highly significantly different from the value predicted from a monogenic model (*P* = 1.61E-08) (S4 Table). Thus, the hypothesis that one distinct gene is responsible for strong resistance compared to weak resistance is not supported by the data.

## Discussion

Previous studies have indicated that alleles of two genes, *rph1* and *rph2*, are responsible for the strong resistance phenotype in *Rhyzopertha dominica* and *Tribolium castaneum*. The identity of the *rph2* resistance factor is known and has been shown to be the same gene in both species, whereas the identity of *rph1* is not known in any species [12]. It is likewise not known whether resistance is due to the same two genes in other major pest species, which provided the motivation to analyse the genetics of phosphine resistance in *S*. *oryzae*, another major pest of grain that exhibits strong resistance to phosphine.

Our analysis of the responses of F_2_ and F_1_-BC progenies of a series of crosses between S-strain (susceptible to phosphine), W-strain (weak resistance phenotype) and R-strain (strong resistance phenotype) led us to the conclusion that strong resistance to phosphine in *S*. *oryzae* is controlled by at least 2 major genes. One of these genes is responsible for weak resistance in the W-strain and contributes to resistance in the R-strain as well. These findings are consistent with results from Li and Li (1994) who concluded that strong resistance in a Chinese strain was controlled by more than one autosomal factor [6]. The results in *S*. *oryzae* are also consistent with results in *R*. *dominica* [13, 26–31] and *T*. *castaneum* [14, 27, 32] in which the gene responsible for weak resistance also contributes to the strong resistance trait. Weak resistance was previously genetically characterised in *S*. *oryzae* [7] and was found to be incompletely recessive, autosomally inherited and lacking any maternal influence. As these characteristics are shared with the *rph1* genes in *T*. *castaneum* and *R*. *dominica*, we will name the weak resistance factor in *S*. *oryzae So_rph1* (*S*. *oryzae_* *r* *esistance to* *ph* *osphine 1*).

The additional factor, that together with *So_rph1* gives rise to the strong resistance phenotype in *S*. *oryzae*, is also incompletely recessive, autosomally inherited and without maternal influence. The mode of inheritance of strong resistance to phosphine in *S*. *oryzae* in our current study is consistent with results of genetic analyses of phosphine resistance on the same species [6, 7] and in two other species of grain pests, *R*. *dominica* [26] and *T*. *castaneum* [14, 32]. Strong resistance is an incompletely recessive, autosomal trait in all isolates of all three species. One distinction is that the resistance factor of the strongly resistant strain in *S*. *oryzae* is less than that of other insect pests when determined from the LC_50_ values for a 48 h exposure. Thus, the strongly resistant strain of *S*. *oryzae* is 52 times more resistant than the basal tolerance of a susceptible reference strain and 9 times the resistance of the weakly resistant strain. In contrast, the resistance factor of the strongly resistant strains of *R*. *dominica* range from 100 to 225 times that of the susceptible reference strain [29], with one report of 600 times [26]. Strong resistance in *T*. *castaneum* is 90 times that of the fully susceptible strain given a 48 h exposure (Jagadeesan, unpublished) but reaches 431 times for a 20 h phosphine exposure [14].

Despite the broad similarity between phosphine resistance in *S*. *oryzae* and other pest insects, we did make an interesting and unique observation. A small proportion of the F_1_-BC progeny was unusually strongly resistant to phosphine, which was observed as a plateau in the probit mortality curve at the highest concentrations tested (Figs 2 and 4), even to the extent that some individuals were more resistant to phosphine than the strongly resistant parent. This result, together with other deviations from a model that assumes that the strong resistance trait can be explained by a single gene in addition to *So_rph1*, has led us to conclude that resistance in the strains we tested is more complex than what has previously been observed in *T*. *castaneum* and *R*. *dominica*.

These results may indicate the presence of multiple additional resistance genes or may be explained by multiple alleles at one or more resistance loci, which differ in the strength of the resistance phenotype they confer. The latter interpretation is supported by the observation that despite being selected multiple times to ensure homozygosity for resistance alleles, the strongly resistant strain of *S*. *oryzae* exhibited phenotypic heterogeneity, unlike the susceptible and weakly resistant strains. Future DNA sequence analysis of the resistance genes may clarify this point. The ambiguity regarding the genetics of strong resistance in *S*. *oryzae* makes it premature to propose the existence of a genetic equivalent of the *rph2* locus previously described for *T*. *castaneum* and *R*. *dominica* [12].

In many ways the genetics of phosphine resistance is similar in each of the pest species studied so far. However, until the molecular identity of the resistance genes is known in each species, our ability to make direct comparisons remains limited. While the identity of the *rph1* gene remains unknown, *rph2* is known to be mediated by the dihydrolipoamide dehydrogenase (*dld*) gene in both *R*. *dominica* and *T*. *castaneum* [12]. As most properties of the resistance trait are similar between those species and *S*. *oryzae*, it is reasonable to predict that the *dld* gene will contribute to phosphine resistance in the latter. If this proves to be the case, a simple molecular tool can be developed to effectively identify resistance alleles and assist with resistance management in *S*. *oryzae* and other stored product insects.

## Supporting Information

S1 Table Chi-square test of the one gene model of phosphine resistance based on the F_2_ progeny of an S-strain x R-strain cross. (DOCX)

S2 Table Chi-square test of the one gene model of phosphine resistance based on the progeny of an F_1_ x R-strain backcross, where the F_1_ was generated from an S-strain (♀) x R-strain (♂) cross. (DOCX)

S3 Table Chi-square test of the one gene model of phosphine resistance based on the F_2_ progeny of a W-strain x R-strain cross. (DOCX)

S4 Table Chi-square test of the one gene model of phosphine resistance based on the progeny of an F_1_ x R-strain backcross, where the F_1_ was generated from a W-strain (♀) x R-strain (♂) cross. (DOCX)

## References

[1] Bell C. Fumigation in the 21st century. Crop Protection. 2000;19(8):563–9.

[2] Chaudhry MQ. Phosphine resistance. Pesticide Outlook. 2000;11(3):88–91.

[3] Scheffrahn RH, Hsu RC, Osbrink WL, Su NY. Fluoride and sulfate residues in foods fumigated with sulfuryl fluoride. Journal of Agricultural and Food Chemistry. 1989;37(1):203–6.

[4] Mills K. Phosphine resistance: where to now. In: Donahaye, EJ, Navarro, S and Leesch JG, editors. Proceeding Internatinal Conference on Controlled Atmosphere and Fumigation in Stored Products; 2000 Oct 29—Nov 3; Fresno, USA. 2000:583–91.

[5] Champ B, Dyte C. FAO global survey of pesticide susceptibility of stored grain pests. FAO Plant Protection Bulletin. 1977;25(2):49–67.

[6] Li Y, Li W. Inheritance of phosphine resistance in *Sitophilus oryzae* (L.) (Coleoptera, Curculionidae). In: E Highley, EJ Wright, HJ Banks and BR Champ, editors. Stored Products Protection: Proceeding of the 6th International Working Conference on Stored-Product Protection, 1994 Apr 17–23, Canberra, Australia CAB International, Wallingford, UK. 1994:113–5.

[7] Daglish GJ, Nayak MK, Pavic H. Phosphine resistance in *Sitophilus oryzae* (L.) from eastern Australia: Inheritance, fitness and prevalence. Journal of Stored Products Research. 2014;59:237–44.

[8] Zeng L. Development and countermeasures of phosphine resistance in stored grain insects in Guangdong of China. In: Jin, Z; Liang, Q; Liang, Y; Tan, X; Guan, L, editors. Proceedings of the 7th International Working Conference on Stored-Product Protection, 1998 Oct 14–19, Beijing, China Sichuan Publishing House of Science and Technology, Chengdu, China, 1999. 1998;1:642–7.

[9] Rajendran S. Phosphine resistance in stored insect pests in India. In: Jin, Z; Liang, Q; Liang, Y; Tan, X; Guan, L, editors. Proceedings of the 7th International Working Conference on Stored-Product Protection, 1998 Oct 14–19, Beijing, China Sichuan Publishing House of Science and Technology, Chengdu, China, 1999. 1998;1:635–41.

[10] Emery RN, Collins JP, Wallbank EB. Monitoring and managing phosphine resistance in Australia. In: Wright, EJ, Webb, MC and Highley, E, editors. Stored Grain in Australia 2003: Proceedings of the Australian Postharvest Technical Conference, 2003 Jun 25–27, Canberra, Australia. 2003:142–51.

[11] Schlipalius D. Diagnostic technologies for phosphine resistance management. Final report for CRC20080 project of Cooperative Research Centre for National Plant Biosecurity, 2010 Jun 30. Available: http://legacy.crcplantbiosecurity.com.au/sites/all/files/20080_final_report.pdf.

[12] Schlipalius DI, Valmas N, Tuck AG, Jagadeesan R, Ma L, Kaur R, et al A Core Metabolic Enzyme Mediates Resistance to Phosphine Gas. Science. 2012;338(6108):807–10. 10.1126/science.1224951 23139334

[13] Schlipalius DI, Cheng Q, Reilly PEB, Collins PJ, Ebert PR. Genetic linkage analysis of the lesser grain borer *Rhyzopertha dominica* identifies two loci that confer high-level resistance to the fumigant phosphine. Genetics. 2002;161(2):773–82. 12072472 10.1093/genetics/161.2.773 PMC1462159

[14] Jagadeesan R, Collins PJ, Daglish GJ, Ebert PR, Schlipalius DI. Phosphine resistance in the Rust Red Flour Beetle, *Tribolium castaneum* (Coleoptera: Tenebrionidae): inheritance, gene interactions and fitness costs. Plos One. 2012;7(2):e31582 10.1371/journal.pone.0031582 22363681 PMC3283673

[15] Daglish GJ, Collins PJ, Pavic H, Kopittke RA. Effects of time and concentration on mortality of phosphine-resistant *Sitophilus oryzae* (L) fumigated with phosphine. Pest Management Science. 2002;58(10):1015–21. 12400440 10.1002/ps.532

[16] Anon. Recommended methods for detection and management of resistance of agricultural pests to pesticide. Tentative method for adults of some major pest species of stored cereals, with methyl bromide and phosphine. FAO Method No.16. FAO Plant Prot Bull. 1975;23:12–26.

[17] Finney DJ. Probit analysis, 3rd edn. University Printing House, Cambridge, England 1971.

[18] Committee G. Genstat Release 11.1. VSN International, Oxford, UK 2008.

[19] Abbott WS. A method of computing the effectiveness of an insecticide. Journal of Economic Entomology. 1925;18:265–7.

[20] Robertson JL, Savin N, Preisler HK, Russell RM. Bioassays with arthropods: CRC press; 2007.

[21] Stone BF. A formula for determining degree of dominance in cases of monofactorial inheritance of resistance to chemicals. Bulletin of the World Health Organization. 1968;38(2):325–6. 5302309 PMC2554319

[22] Georghiou GP. Genetics of resistance to insecticides in houseflies and mosquitoes. Experimental Parasitology. 1969;26(2):224–55. 4927150 10.1016/0014-4894(69)90116-7

[23] Preisler HK, Hoy MA, Robertson JL. Statistical analysis of modes of inheritance for pesticide resistance. Journal of Economic Entomology. 1990;83(5):1649–55.

[24] Tsukamoto M. The log dosage-probit mortality curve in genetic researches of insect resistance to insecticides. Botyu-Kagaku. 1963;28(4):91–8.

[25] Tabashnik BE. Determining the mode of inheritance of pesticide resistance with backcross experiments. Journal of Economic Entomology. 1991;84(3):703–12. 1885840 10.1093/jee/84.3.703

[26] Collins PJ, Daglish GJ, Bengston M, Lambkin TM, Pavic H. Genetics of resistance to phosphine in *Rhyzopertha dominica* (Coleoptera: Bostrichidae). Journal of Economic Entomology. 2002;95(4):862–9. 12216832 10.1603/0022-0493-95.4.862

[27] Ansell M, Dyte C, Smith R. The inheritance of phosphine resistance in *Rhyzopertha dominica* and *Tribolium castaneum*. In: Fleurat-Lessard, F; Ducom, P, editors. Proceedings of the 5th International Working Conference on Stored-Product Protection, 1990 Sep 9–14, Bordeaux, France Imprimerie du Médoc, Bordeaux, France, 1991. 1990:961–9.

[28] Li YS, Li WZ, Li WW, Wu XQ. Genetic analysis of phosphine resistance in *Rhyzopertha dominica* and *Sitophilus oryzae* . Acta entomologica Sinica 1994;37:271–9.

[29] Mau YS, Collins PJ, Daglish GJ, Nayak MK, Pavic H, Ebert PR. The rph1 Gene Is a Common Contributor to the Evolution of Phosphine Resistance in Independent Field Isolates of *Rhyzopertha dominica* . Plos One. 2012;7(2):e31541 10.1371/journal.pone.0031541 22363668 PMC3282749

[30] Mau YS, Collins PJ, Daglish GJ, Nayak MK, Ebert PR. The rph2 gene is responsible for high level resistance to phosphine in independent field strains of *Rhyzopertha dominica* . Plos One. 2012;7(3):e34027 Epub 2012/03/31. 10.1371/journal.pone.0034027 22461899 PMC3312893

[31] Kaur R, Schlipalius DI, Collins PJ, Swain AJ, Ebert PR. Inheritance and relative dominance, expressed as toxicity response and delayed development, of phosphine resistance in immature stages of *Rhyzopertha dominica*(F.)(Coleoptera: Bostrichidae). Journal of Stored Products Research. 2012;51:74–80.

[32] Bengston M, Collins PJ, Daglish GJ, Hallman VL, Kopittke RM, Pavic H. Inheritance of phosphine resistance in *Tribolium castaneum* (Coleoptera: Tenebrionidae). Journal of Economic Entomology. 1999;92(1):17–20.

