# Supplementary material for: Inheritance and Characterization of Strong Resistance to Phosphine in Sitophilus oryzae (L.)
Source: PLoS One. 2015 Apr 17;10(4):e0124335. doi: 10.1371/journal.pone.0124335 (PMC4401577; doi:10.1371/journal.pone.0124335)
Supplement: S1 Table — (DOCX) [file pone.0124335.s001.docx]

**S1 Table. Chi-square test of the one gene model of phosphine resistance based on the F_2_ progeny of an S-strain x R-strain cross.**

|  |  | **Mortality (number)** | |  |  |
| --- | --- | --- | --- | --- | --- |
| **Dose (mg L^-1^)** | **n** | **Observed** | **Expected** | **Modified χ^2^** | **P** |
| 0.005 | 854 | 123 | 139.8 | 0.206 | 0.650 |
| 0.008 | 862 | 281 | 333.7 | 1.161 | 0.281 |
| 0.01 | 855 | 417 | 424.5 | 0.023 | 0.880 |
| 0.015 | 851 | 557 | 552.6 | 0.009 | 0.926 |
| 0.03 | 856 | 685 | 635.6 | 1.274 | 0.259 |
| 0.06 | 899 | 848 | 674.7 | 15.234 | 9.5E-05*** |
| 0.1 | 902 | 868 | 683.5 | 17.556 | 2.79E-05*** |
| 0.3 | 902 | 886 | 802.1 | 6.764 | 0.0093** |
| 0.4 | 905 | 895 | 848.1 | 3.524 | 0.061 |

n = number of insects tested; χ^2^ = chi-square; P = probability value. Expected = number of dead insects expected based on a one gene model of phosphine resistance. Weighted mean heterogeneity factor = 11.71. *Significant (P<0.05); **Significant (P<0.01); ***Significant (P<0.001).
