# Supplementary material for: Inheritance and Characterization of Strong Resistance to Phosphine in Sitophilus oryzae (L.)
Source: PLoS One. 2015 Apr 17;10(4):e0124335. doi: 10.1371/journal.pone.0124335 (PMC4401577; doi:10.1371/journal.pone.0124335)
Supplement: S2 Table — (DOCX) [file pone.0124335.s002.docx]

**S2 Table. Chi-square test of the one gene model of phosphine resistance based on the progeny of an F_1_ x R-strain backcross, where the F_1_ was generated from an S-strain (♀) x R-strain (♂) cross.**

|  |  | **Mortality (number)** | |  |  |
| --- | --- | --- | --- | --- | --- |
| **Dose (mg L^-1^)** | **n** | **Observed** | **Expected** | **Modified χ^2^** | **P** |
| 0.005 | 301 | 3 | 19.1 | 1.153 | 0.283 |
| 0.007 | 300 | 17 | 44.9 | 1.631 | 0.202 |
| 0.008 | 402 | 38 | 78.3 | 2.054 | 0.152 |
| 0.01 | 401 | 49 | 110.5 | 3.770 | 0.052 |
| 0.015 | 304 | 52 | 122.8 | 5.476 | 0.019* |
| 0.02 | 253 | 90 | 116.3 | 0.876 | 0.349 |
| 0.025 | 251 | 104 | 121.1 | 0.371 | 0.542 |
| 0.03 | 401 | 167 | 197.3 | 0.731 | 0.393 |
| 0.04 | 251 | 136 | 125.0 | 0.153 | 0.696 |
| 0.05 | 250 | 143 | 125.0 | 0.415 | 0.519 |
| 0.06 | 400 | 273 | 200.4 | 4.204 | 0.040* |
| 0.08 | 250 | 187 | 126.4 | 4.688 | 0.030* |
| 0.1 | 400 | 300 | 206.2 | 7.032 | 0.008** |
| 0.3 | 298 | 271 | 232.0 | 2.363 | 0.124 |
| 0.4 | 402 | 377 | 351.4 | 1.180 | 0.277 |
| 0.6 | 304 | 289 | 292.0 | 0.061 | 0.805 |
| 0.9 | 400 | 380 | 396.9 | 7.298 | 0.007** |

n = number of insects tested; χ^2^ = chi-square; P = probability value. Expected = number of dead insects expected based on a one gene model of phosphine resistance. Weighted mean heterogeneity factor = 12.52. *Significant (P<0.05); **Significant (P<0.01); ***Significant (P<0.001).
