# Supplementary material for: Inheritance and Characterization of Strong Resistance to Phosphine in Sitophilus oryzae (L.)
Source: PLoS One. 2015 Apr 17;10(4):e0124335. doi: 10.1371/journal.pone.0124335 (PMC4401577; doi:10.1371/journal.pone.0124335)
Supplement: S3 Table — (DOCX) [file pone.0124335.s003.docx]

**S3 Table. Chi-square test of the one gene model of phosphine resistance based on the F_2_ progeny of a W-strain x R-strain cross.**

|  |  | **Mortality (number)** | |  |  |
| --- | --- | --- | --- | --- | --- |
| **Dose (mg L^-1^)** | **n** | **Observed** | **Expected** | **Modified χ^2^** | **P** |
| 0.02 | 913 | 69 | 65.8 | 0.023 | 0.880 |
| 0.03 | 909 | 148 | 155.8 | 0.062 | 0.804 |
| 0.05 | 909 | 436 | 351.8 | 4.357 | 0.037* |
| 0.08 | 908 | 625 | 546.9 | 4.050 | 0.044* |
| 0.15 | 912 | 791 | 697.5 | 7.061 | 0.008** |
| 0.2 | 904 | 822 | 736.1 | 7.140 | 0.008** |
| 0.3 | 904 | 809 | 803.6 | 0.044 | 0.834 |
| 0.5 | 909 | 893 | 877.0 | 1.093 | 0.296 |
| 0.6 | 912 | 912 | 894.0 | 2.437 | 0.119 |

n = number of insects tested; χ^2^ = chi-square; P = probability value. Expected = number of dead insects expected based on a one gene model of phosphine resistance. Weighted mean heterogeneity factor = 7.55.*Significant (P<0.05); **Significant (P<0.01); ***Significant (P<0.001).
