# Supplementary material for: Inheritance and Characterization of Strong Resistance to Phosphine in Sitophilus oryzae (L.)
Source: PLoS One. 2015 Apr 17;10(4):e0124335. doi: 10.1371/journal.pone.0124335 (PMC4401577; doi:10.1371/journal.pone.0124335)
Supplement: S4 Table — (DOCX) [file pone.0124335.s004.docx]

**S4 Table. Chi-square test of the one gene model of phosphine resistance based on the progeny of an F_1_ x R-strain backcross, where the F_1_ was generated from a W-strain (♀) x R-strain (♂) cross.**

|  |  | **Mortality (number)** | |  |  |
| --- | --- | --- | --- | --- | --- |
| **Dose (mg L^-1^)** | **n** | **Observed** | **Expected** | **Modified χ^2^** | **P** |
| 0.03 | 350 | 8 | 18.5 | 0.815 | 0.367 |
| 0.06 | 353 | 102 | 94.4 | 0.109 | 0.741 |
| 0.08 | 407 | 173 | 152.2 | 0.587 | 0.444 |
| 0.1 | 403 | 185 | 178.6 | 0.053 | 0.819 |
| 0.15 | 402 | 274 | 220.6 | 3.698 | 0.054 |
| 0.2 | 402 | 329 | 254.6 | 7.659 | 0.006** |
| 0.3 | 401 | 373 | 312.0 | 6.930 | 0.008** |
| 0.4 | 385 | 334 | 336.6 | 0.020 | 0.888 |
| 0.6 | 402 | 377 | 386.1 | 0.699 | 0.403 |
| 0.9 | 403 | 372 | 399.8 | 31.914 | 1.61E-08*** |

n = number of insects tested; χ^2^ = chi-square; P = probability value. Expected = number of dead insects expected based on a one gene model of phosphine resistance. Weighted mean heterogeneity factor = 7.75. *Significant (P<0.05); **Significant (P<0.01); ***Significant (P<0.001).
